# Temperature Dependence of Electronic Transport Mechanisms in rGO-Based Photodetectors

**DOI:** 10.3390/nano16040222

**Published:** 2026-02-07

**Authors:** Carmela Bonavolontà, Antonio Vettoliere, Berardo Ruggiero, Carmine Granata, Massimo Valentino

**Affiliations:** CNR-ISASI, Institute of Applied Sciences and Intelligent Systems “Eduardo Caianiello”, Via Campi Flegrei 34, I-80078 Pozzuoli, Italy; antonio.vettoliere@isasi.cnr.it (A.V.); berardo.ruggiero@isasi.cnr.it (B.R.); carmine.granata@isasi.cnr.it (C.G.); massimo.valentino@isasi.cnr.it (M.V.)

**Keywords:** photodetectors, reduced graphene oxide, heterojunction, cryogenic temperature, quantum tunnel

## Abstract

Reduced graphene oxide (rGO) has attracted interest as a potential, cost-effective alternative to graphene layers produced by single-crystal thin-film growth techniques. Its solubility in various solvents, the ability to tune its optical and electrical properties, the ability to manipulate the optoelectronic properties of rGO-based heterojunctions, and the possibility of depositing it on flexible substrates broaden its potential applications, from electro-optical communications to environmental monitoring. In this work, we present a characterization of reduced graphene oxide (rGO) deposited on p-type Si_3_N_4_/Si substrate using different techniques such as Raman spectroscopy, optical transmittance, and current-voltage measurements under dark and illuminated conditions in the 400–700 nm range. Furthermore, the temperature dependence of the photocurrent of the rGO-based photoconductive device was studied in the temperature range from 300 K to 77 K. It has been shown that the electron transport mechanism through the p-type rGO/SiN/Si heterojunction at low voltage involves mainly a hopping process at 77 K and a thermionic mechanism at room temperature. Furthermore, the Fowler–Nordheim tunneling and trap-limiting mechanisms allow the presence of charge carriers in the device at both temperatures. Estimation of the main figures of merit, responsivity, detectivity, and NEP, shows an improvement in photodetection performance at low temperatures.

## 1. Introduction

Reduced graphene oxide (rGO) is of interest from a materials science perspective [1,2,3]. Thanks to its abundance of oxygen-containing functional groups and the controllable ratio of sp2- and sp3-hybridized carbon atoms, rGO possesses tunable electronic and optical properties, opening exciting new applications in various fields. For example, its tunable band gap makes rGO suitable for electrical and optical devices, including electrical sensors, field-emission devices, photovoltaic devices, and photodetectors. The presence of functional groups in GO and rGO [4,5] makes them readily compatible with other nanomaterials, such as nanoparticles, carbon nanotubes, and conductive polymers. GO, or its reduced form (rGO), and their composites show great potential in energy storage/conversion and environmental protection technologies, such as photocatalysts for water splitting under solar irradiation, or as carriers for hydrogen storage, as well as electrodes for various lithium batteries, supercapacitors, and for removing pollutants from air and water [6].

Furthermore, the functional groups make GO hydrophilic and soluble, and they interact non-covalently with biomolecules. Therefore, GO is useful in biotechnology for detecting biomolecules with high sensitivity and specificity, as well as for drug delivery. In principle, there are nearly unlimited combinations of GO and rGO with other functional nanomaterials, making composites a largely unexplored field with many potential applications [7,8,9,10,11,12,13,14,15,16,17,18,19]. It is essential to understand the interaction mechanism between GO and rGO at the nanoscale from an atomistic perspective. From this perspective, further investigations into the intrinsic synergistic effects of GO and rGO composites in improving overall energy storage and conversion performance, environmental protection applications, and biotechnology are warranted [20,21,22]. Concerning the sensors useful for photoelectric conversion, they are at the center of numerous technological and scientific applications such as imaging, optical communication, solar energy, energy harvesting, photoelectric memories, military reconnaissance, and astronomical studies [23,24,25].

Traditionally, photodetectors (PDs) were mainly developed for ultraviolet (UV), visible (Vis), near-infrared (NIR), mid-infrared (MIR), and far-infrared (FIR) applications by using different semiconductors like GaN, Si, Ge, InGaAs, and HgCdTe, each specifically suited to absorb a particular range of wavelengths [26,27,28]. Silicon has been widely used for the fabrication of CMOS image sensors, which have made a significant impact on the markets for camcorders and mobile phones. Unfortunately, Si has low efficiency in both UV and NIR spectra. For UV, this is mainly due to the low penetration depth of UV radiation, which is absorbed near the Si interface, where surface recombination is highest. In the NIR, silicon’s absorption decreases sharply with increasing wavelength and can be neglected beyond 1100 nm, given its bandgap of 1.12 eV. Additionally, PDs based on silicon’s conventional heterojunctions (HJPDS) often suffer from numerous interfacial defects, leading to poor carrier separation, low efficiency, and weak light absorption, severely limiting their photoelectric detection capabilities [25,29].

The performance of light detectors is being improved by new materials that replace traditional silicon-based materials in PDs. Graphene is one of the most promising 2D materials thanks to its electrical, mechanical, and thermal properties, making it suitable for solar cells, field-effect transistors, sensors, and photodetectors [24,25]. Heterointerfaces formed by 2D materials can alter the optical properties of dissimilar materials [30,31], facilitating band alignment and optical interactions between them, and improving the effective spatial separation of electron–hole pairs and the tunneling efficiency [32]. The best device performance is achieved with junctions made on a silicon substrate using graphene and its derivatives, such as graphene oxide, reduced graphene oxide, and carbon nanotubes [33]. The recent results obtained with the rGO/n-Si device demonstrate a value of responsivity (0.2 A/W around 650 nm), close to the values reported for commercial Si PDs, and indicate potential for a broadband device [34,35,36]. The detection mechanism is probably tunneling in metal–oxide–semiconductor (MOS) structures, and device performance is limited by the optical transmittance of the active material (rGO). rGO provides superior charge-carrier mobility, enabling further improvement in the performance of visible-light photodetectors and in memory storage and logic applications [37]. rGO is an electrically semi-conductive material similar to disordered graphene; it displays weak gate-voltage sensitivity (on/off ratio < 10) and is therefore being investigated as a transparent conductor replacement for indium tin oxide (ITO) [7,8]. rGO has been shown to enhance the performance of devices requiring solution processing, mechanical flexibility, and electrochemical stability [10,11,12,38].

Broadband photodetectors are light-sensitive devices that can operate at very low temperatures to improve performance in terms of sensitivity, quantum efficiency, signal-to-noise ratio, and reduced dark counts. These are all essential for astronomical and space applications [39,40,41,42,43]. Photodetectors are typically used in low-temperature environments because low temperatures effectively suppress dark current and background noise, thereby improving detection performance. However, higher operating temperatures can cause thermal excitation, leading to noise interference, reduced detector performance, and a sharp deterioration in the imaging quality of the target object. Therefore, testing the performance of the photodetectors at low temperatures is useful for a better understanding of their applicability in such environmental conditions.

In this work, the photogeneration performance of a MOS structure based on rGO is investigated at room temperature and 77 K across the incident-light spectrum from 400 nm to 700 nm. At this cryogenic temperature, the thermionic contribution to the charge-transport mechanism is drastically reduced; consequently, the quantum processes involved in photodetection are highlighted. The temperature-dependent mechanism investigation reported in this work is a key tool for understanding the critical parameters governing MOS structures, such as the rGO/Si_3_N_4_/Si heterojunction, and their potential role in astronomical photodetection applications. To this aim, the main factors of merit of the device, such as detectivity, NEP (noise equivalent power), and responsivity, were measured at room temperature and critically compared with the measurements obtained at cryogenic temperature.

## 2. Materials and Methods

Graphene oxide (GO) was synthesized from graphite powder using a modified Hummers method. The GO solution (4% in distilled water purchased from Graphenea Inc., (Cambridge, MA 02142, USA) was treated with ultrasound for about an hour to eliminate potential clusters. The solution was then deposited on the substrate by spin coating and thermally reduced to form an rGO layer with a thickness of 0.1 mm. Further details about the fabrication process have already been reported in ref. [35]. The rGO area was defined using an adhesive mask; in this case, a 5 × 8 mm^2^ rGO area was obtained, leaving a 40 mm^2^ photodetector active area.

A p-type silicon wafer with a thickness of 300 μm and a resistivity of 8–12 Ω∙cm was used as the substrate. The p-Si wafer was coated with a 60 nm-thick Si_3_N_4_ layer deposited via plasma-enhanced chemical vapor deposition (PECVD). Two circular Pt/Ti electrodes, each 1 mm in diameter, were deposited on top of the silicon nitride. Additionally, a p+ doped layer was inserted between the silicon and the Pt/Ti back electrode to ensure an Ohmic contact. Finally, a layer of rGO was deposited on the Si_3_N_4_ layer.

**Figure 1:**
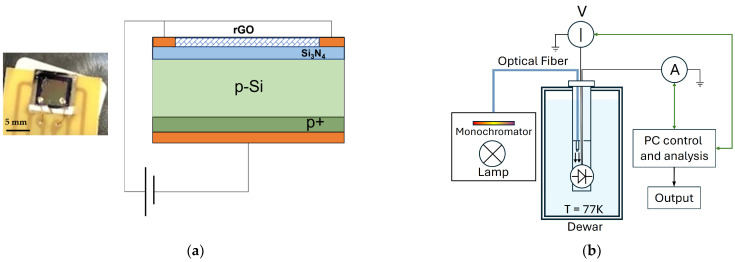
(**a**) Optical image (**left**) and stacking sequence (**right**) of the device, from top to bottom: the rGO layer with Pt/Ti electrodes on the sides, the Si_3_N_4_ layer with a thickness of 60 nm, the p-type silicon substrate, the p+ doped layer, and the Pt/Ti back electrode. External electrical circuits supply the heterojunction in reverse bias. (**b**) Experimental setup for electro-optical characterization at 77 K and 300 K.

Figure 1a shows the optical image, the device stacking sequence, along with the voltage bias used to measure the current–voltage (I–V) characteristics. This setup allowed us to observe the current flowing vertically across the heterojunction.

The Raman spectra were recorded with a confocal Raman microspectrometer (XploRA PLUS, HORIBA Advanced Techno, Kyoto, Japan) using a 532 nm diode laser at 90 mW. The spectra were collected with a 1 s exposure over the Raman-shift range of 1100–3200 cm^−1^. The UV–Vis–IR transmittance spectrum of the rGO thin film on a glass substrate was measured using a PerkinElmer Lambda 2 spectrometer (Shelton, CT 06484-4794, USA).

The current-voltage characterization at 77 K, in the dark and under illumination, was performed using the system shown in Figure 1b. The device was placed in a dewar containing liquid nitrogen and illuminated via a cryogenic optical fiber (operating in the UV-NIR range, with a working temperature up to 1.5 K, produced by SEDI-ATI (91080 Evry-Courcouronnes, France) positioned very close to the device surface. The optical fiber was positioned perpendicular to the device’s surface to project a light spot with a diameter of about 2 mm. The power light calibration was performed at room temperature by placing the probe in the same position as the sample. The same system was used to measure the I–V at room temperature, but with an empty dewar. The light source for the photoresponse measurements was an Oriel 77501 Fiber Optic Illuminator (MKS Inc. Andover, 01810, MA, USA) with a 100-Watt quartz halogen lamp, operating over 400 nm to 700 nm, with power adjusted from 1 to 15 μW. The I–V characteristics were recorded using a voltage supply (Keithley SourceMeter, model 2635 (Giakova srl. 20019—Settimo Milanese (MI), Italy)) and a dual-channel picoammeter (Keithley, model 6482 (Giakova srl. 20019—Settimo Milanese (MI), Italy)).

## 3. Results

### 3.1. Reduced Graphene Oxide Characterization

**Figure 2:**
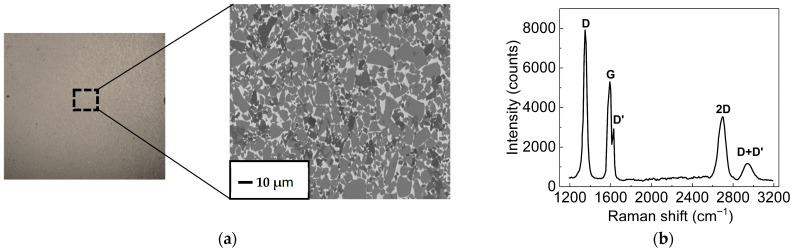
(**a**) Optical image of the reduced graphene oxide layer deposited on a glass substrate. The area inside the rectangular black dashed line is shown as an SEM image to display the flakes covering the rGO layer. (**b**) Raman spectrum of the rGO layer.

The rGO layer deposited on a glass substrate was initially characterized using optical analysis to evaluate its surface structure. In Figure 2a, the optical image of the surface displays the typical dark-brown color resulting from the reduction in graphene oxide [44]. An area measuring 100 × 100 μm^2^ is magnified and shown as an SEM image to illustrate that the rGO surface is covered with flakes of different sizes, up to about 10 × 10 μm^2^. Additionally, Raman analysis was conducted and is presented in Figure 2b.

The Raman spectra of the rGO sample show typical features: the G-band at 1590 cm^−1^ and a 2D-band at 2685 cm^−1^, caused by a second-order two-phonon process. Along with the 2D- and G-bands, peaks also appear at 1345 cm^−1^ (D band) and 1620 cm^−1^ (D′ band). The D peak relates to breathing modes of sp^2^ carbon rings, while the D′ peak indicates in-plane longitudinal optical (LO) phonons at the K point [45]. The presence of both D and D′ peaks suggests defects in the rGO layer, such as bond angle disorder, vacancies, or edge defects [46]. Additionally, their combined peak (D + D′) near 2940 cm^−1^ further supports the presence of a highly defective graphitic structure [45].

It is established that the I_D_/I_G_ ratio indicates the level of defects in rGO samples, and the I_2D_/I_G_ ratio reflects the recovery of the sp^2^ C=C bonds in the graphitic structure, providing insights into the layer arrangement [47]. The calculated values of I_D_/I_G_ and I_2D_/I_G_ are 1.5 and 0.6, respectively. These values suggest a high level of graphitic defects and that the transferred rGO is multilayer [48,49,50,51].

**Figure 3:**
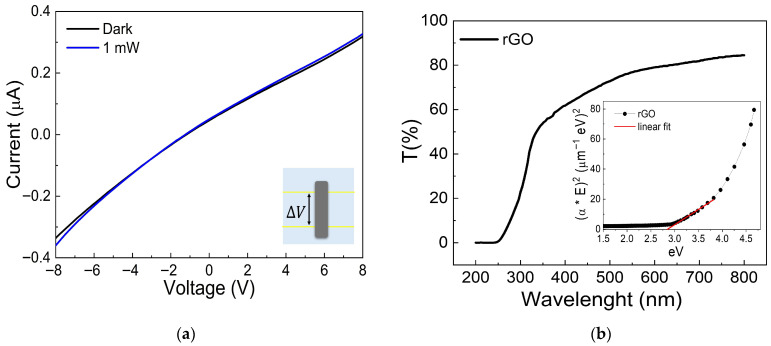
(**a**) I–V characteristics of the rGO layer on a glass substrate in dark and under illumination conditions with a wavelength of 440 nm at 1 mW. In the inset, the device used to measure the I–V is shown, consisting of a glass substrate (the largest rectangular area), two yellow ITO lines spaced 30 mm apart as electrodes where the voltage bias ΔV is applied, and the rGO layer represented by the gray rectangular area. (**b**) The percentage of optical transmittance of the rGO layer. (Inset) Tauc’s plot analysis was used to estimate the bandgap of rGO. The red lines indicate the fitting analysis used to determine the band gap.

In Figure 3a, the I–V curves of the rGO layer, deposited on a glass substrate with two ITO lines (yellow lines in the inset) serving as electrodes, exhibit non-linear behavior, indicating a semiconductor-like material with an electrical resistance of approximately 150 kΩ. Under illumination with 1 mW of 440 nm laser light, a slight increase in current is observed. This results from rGO’s ability to absorb light and generate charge carriers. The transmittance spectrum shown in Figure 3b confirms this, with higher absorption in the 300–500 nm range compared to the visible and NIR regions. Additionally, Tauc’s plot analysis (shown in the inset of Figure 3b) indicates a bandgap of about 2.8 eV for the rGO layer, consistent with its photosensitivity under 440 nm illumination, as evidenced by the increased photocurrent in Figure 3a.

### 3.2. Temperature Characterization of rGO/Si_3_N_4_/p-Si-Based Photodetector

This section presents the characterization of the rGO/Si_3_N_4_/p-Si device. It includes analysis conducted in dark conditions and under illumination at low temperature (77 K) and room temperature (300 K).

**Figure 4:**
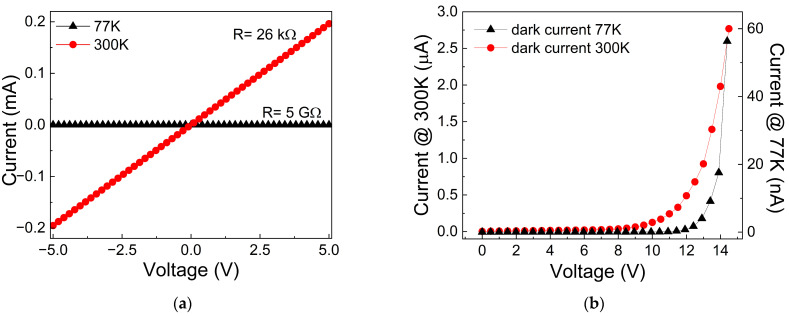
(**a**) I–V characterization of the rGO layer deposited on the Si_3_N_4_/p-Si substrate measured at 77 K and 300 K; (**b**) dark current of the rGO/Si_3_N_4_/p-Si device at temperatures of 77 K and 300 K.

The current-voltage curve measured in the dark between the two electrodes on the device surface provides an estimate of the electrical resistance of the rGO layer deposited on the Si_3_N_4_/p-Si substrate. As shown in Figure 4a, and as expected, the electrical resistance of the rGO layer increases significantly at low temperature, confirming the semiconducting nature of the rGO in agreement with the literature [52]. On the other hand, at 300 K, the rGO resistance of about 26 kΩ is lower than that observed in the I–V curve shown in Figure 3a for the rGO deposited on the glass substrate. This difference could be attributed to the smaller distance (about 5 mm) between the electrodes through which the rGO is included when deposited on the Si-based substrate. However, the contribution of the traps present in the rGO flake-like layer cannot be completely excluded. As discussed in the next paragraph, charge transport across the heterojunction is influenced by the trap-based mechanism (i.e., the Fowler–Nordheim mechanism), which contributes to reducing the charge recombination process and enhancing the photocurrent.

The dark current of the device at 77 K and 300 K was estimated, as reported in Figure 4b. It can be noted that at 77 K, the dark current is significantly lower than at 300 K, and the curve’s trend is slightly different: the current increase occurs at different voltage biases. Generally, the dark current plays a crucial role in photodetection efficiency; the lower the dark current, the higher the photogeneration.

To examine this feature, the photodetection ability of the rGO-based device was tested by measuring the I–V curve across various wavelengths from 400 nm to 700 nm and at temperatures of 77 K and 300 K. Notably, at both temperatures, the device exhibits maximum photocurrent at 700 nm, while the voltage threshold (Vth) varies with temperature. Specifically, at 77 K, the Vth is around 3 V, but at 300 K, it disappears, and the photocurrent begins to rise rapidly at a few millivolts. This behavior is likely related to the dominant transport mechanism at room temperature, which is mainly thermionic. Therefore, the barrier height of the heterojunction is more easily overcome by charge carriers at room temperature than at 77 K, where it is reasonable to ignore thermionic contributions in favor of tunneling, which requires a specific electric field.

**Figure 5:**
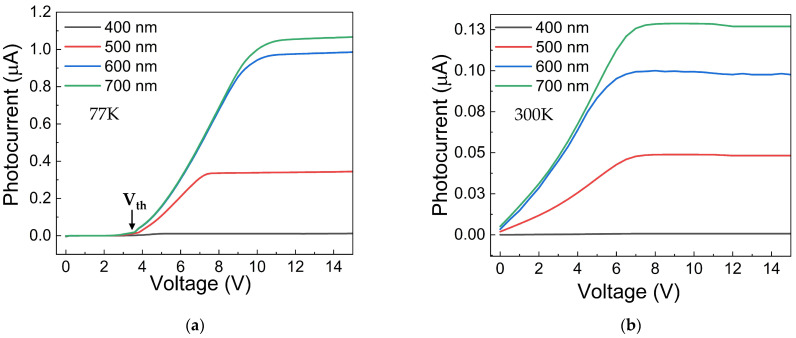
Photocurrent of the rGO/Si_3_N_4_/p-Si device under illumination at wavelengths of 400 nm, 500 nm, 600 nm, and 700 nm, with a power of 15 μW at temperatures of (**a**) 77 K and (**b**) 300 K.

**Figure 6:**
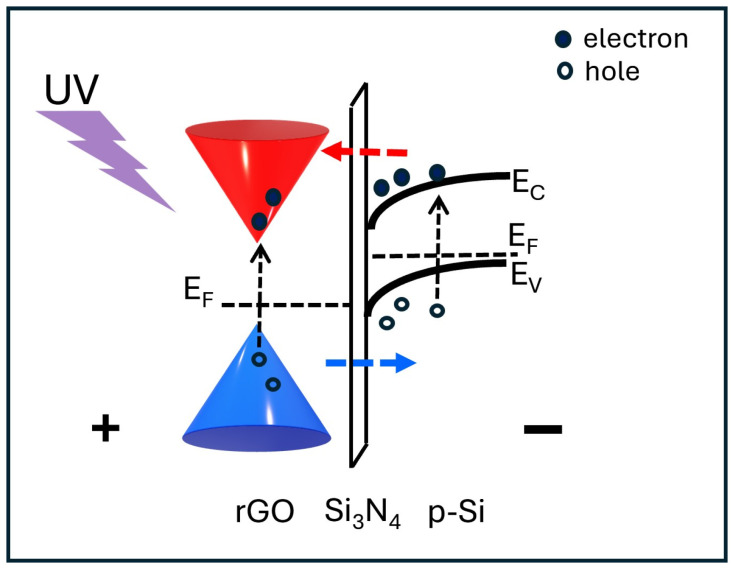
Energy band diagrams of the rGO/Si_3_N_4_/p-Si heterojunctions in reverse bias configuration (+ and − indicate the applied bias across the heterojunction). Under UV illumination, electron–hole pairs are generated in the rGO layer and in the silicon depletion layer (black dashed arrows). The applied field separates the charges: electrons move toward the positive electrode (red dashed arrow) and holes toward the negative electrode (blue dashed arrow).

Additionally, the threshold voltage Vth of the photocurrent-voltage curve signifies the point at which charge carriers cross the heterojunction barrier. This occurs only when the electric field is strong enough to enable tunneling. It is important to note that this process depends solely on the heterojunction properties—such as barrier height, depletion region, and built-in potential—and not on wavelength. Conversely, the thermionic mechanism facilitates charge transfer across the heterojunction but also increases thermal noise and charge recombination. Consequently, the photocurrent at 300 K is lower than at 77 K because, with higher thermal energy (∝ K_B_T), charges (electron–hole pairs) recombine more easily at 300 K than at 77 K, so the photogenerated charges cannot contribute to the photocurrent. Then, the I–V curves in Figure 5 indicate that the rGO-based device exhibits a significant increase in photocurrent at low temperatures, enabling substantial charge-carrier generation across the entire wavelength range explored. In particular, the contribution of rGO as a photoactive material is restricted to the UV range (approximately 440 nm), as demonstrated by the Tauc’s plot estimation of the optical bandgap (Figure 3b). The photodetection mechanism under UV radiation is described in the energy band diagram reported in Figure 6. Instead, the photocurrent in the VIS-NIR range is mainly due to the generation of electron–hole pairs in the heterojunction depletion region (i.e., in the silicon layer across the interface) and their efficient separation [35]. The rGO layer primarily facilitates efficient carrier extraction in the VIS-NIR spectrum, reducing recombination losses through its high carrier mobility and enhancing carrier separation.

The combination of rGO’s conductive and light-absorption properties provides an active interfacial layer that influences the separation of photogenerated carriers and enhances the built-in potential of the rGO/p-Si heterojunction [9,53,54]. Therefore, in the device under test, the rGO serves as both a transparent electrode for light illumination (mainly in VIS-NIR range) and an active layer for electron–hole separation and charge transport [10,11,55,56].

## 4. Discussion

The results shown earlier indicate that the device’s photodetection performance varies with temperature. The experimental data demonstrate that the photocurrent at 77 K is higher than at room temperature. In contrast, the dark current shows the opposite pattern: at 77 K, the device exhibits a lower dark current than at 300 K. Generally, the lower the dark current, the higher the photodetection performance, because a low dark current indicates reduced leakage and recombination. Because photodetection performance is strongly dependent on dark current, it is helpful to analyze the dark current by investigating the device’s transport mechanism to explain the experimental results.

**Figure 7:**
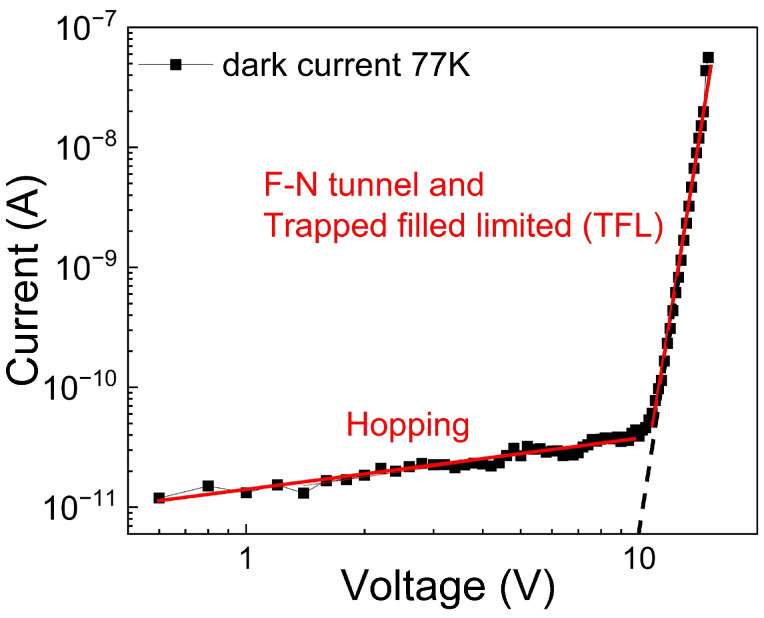
Dark current measured at 77 K. The red lines indicate the linear fit related to the power law of the transport mechanisms present in the two voltage regions, and the dotted black line indicates the estimated transition voltage V_trans_ = 10 V.

To this end, the temperature dependence of the current-voltage curve is a useful tool for discriminating between the different transport mechanisms involved in the heterojunction, such as tunneling and thermionic processes. To sufficiently reduce the thermionic contribution to heterojunction charge transport, the current-voltage curve is measured at 77 K. The log–log plot of current versus applied voltage under reverse bias at 77 K is shown in Figure 7. It is worth noting that there are two regions separated by a transition bias voltage V_trans_ = 10 V derived by the position of the inflection point (dashed black line) in Figure 7.

**Figure 8:**
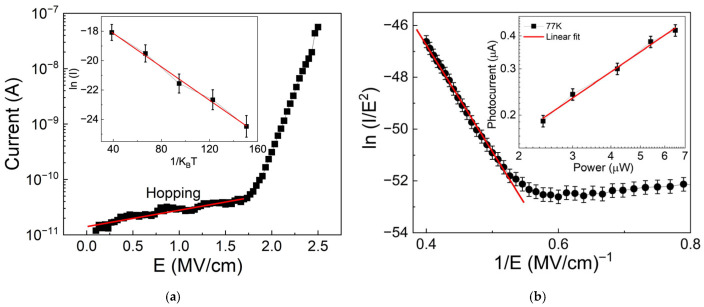
(**a**) Hopping model with a linear fit (red line) used to estimate the trap spacing of about 1.7 nm, (inset) Arrhenius plot used to estimate the hopping activation energy of about 57 meV. (**b**) Fowler–Nordheim model, (inset) photocurrent vs. light power, and the linear fit with a slope of 0.7.

The linear relationship between ln(*I*) and *E* (electric field), with a slope less than one, shown in Figure 8a, indicates that a hopping conduction mechanism is involved. Thanks to this mechanism, which results from tunneling of trapped carriers between trap sites, charges can still move even when their energy is below the potential barriers of the heterojunction. It is reasonable to assume the presence of traps in both the insulator and rGO layers, given their thickness and flake-like structures, respectively.

From the slope of the ln(*I*) versus *E* plot, the average hopping distance was estimated to be about 1.7 ± 0.3 nm, consistent with the hopping distance reported in the literature for the graphene oxide layer [57,58]. Additionally, the activation energy needed for the hopping process was estimated from the slope of the Arrhenius plot of ln(*I*) versus 1/(*K_B_T*), shown in the inset of Figure 8a. The value obtained is approximately 57 ± 2 meV.

Regarding the analysis of the dark current in the region where V > V_trans_, which has a different slope from that in the region where V < V_trans_, ln(*I*/*E*^2^) is plotted against 1/*E* in Figure 8b. The linear dependence observed in the area with a negative slope could be attributed to the Fowler–Nordheim tunneling mechanism. This process occurs when the electric field is strong enough to allow charge carriers to penetrate the triangular heterojunction barrier. Using the relation between the linear fit slope and the barrier height [59,60,61],

(1) $$slope=b\cdot \sqrt{m^{*}{\times \varphi }_{B}^{3}}$$

in which *b* = 6.83∙10^7^ and m* = 0.4 is the effective mass of electrons inside the Si_3_N_4_. The barrier height for the F-N model is estimated to be about 0.9 ± 0.1 eV.

Moreover, in Figure 8, the dark current at V > V_trans_ shows a slope greater than two, which could be attributed to the trap-filled limit (TFL) mechanism, in which the injected carriers are just enough to fill the trap states, leading to a rapid increase in current. To confirm the presence of traps, the variation in the photocurrent with light intensity was studied using the following relation [62].

(2) $$I_{Ph}\;{\propto P}^{m}$$

where *I_Ph_* is the photocurrent, *m* is an exponent, and *P* is the illumination intensity. The value of *m* was determined from the slope of the log (*I_Ph_*)–log(*P*) plot and was 0.7 ± 0.02. The obtained *m* value, between 0.5 and 1, indicates the presence of a continuous distribution of trap levels [63,64,65].

**Figure 9:**
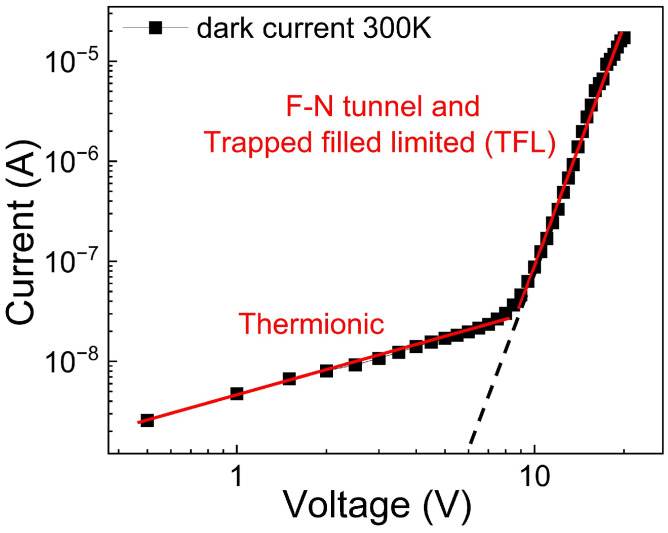
Dark current measured at 300 K, the red lines indicate the transport mechanisms present in the two voltage regions, and the dotted black line indicates the estimated transition voltage V_trans_ = 6 V.

As shown in Figure 4b, the dark current at 300 K and 77 K exhibits different behaviors, mainly at low voltage. To analyze this characteristic, the log–log plot of current versus voltage at 300 K is shown in Figure 9. Similarly, the I–V curve reveals two regions, with the transition occurring at approximately 6 V, as indicated in Figure 9 by the intersection of the dashed line with the voltage axis. Notably, in the region V < V_trans,_ linear dependence with a slope of 0.5 suggests the presence of the Thermionic emission (TE) mechanism. The implication of this mechanism is supported by the comparison of the thermal energy with the parameter defined as $E_{00}=\frac{\hbar q}{2}\sqrt{\frac{N}{m^{*}\varepsilon }}$ where q, ℏ, N, m* and ε is the elementary charge, the Planck constant, the doping concentration of the substrate (10^22^ m^−1^), the charge effective mass, and the silicon dielectric constant, respectively [66,67,68]. E_00_ represents the tunneling characteristic energy and is related to the tunnel probability. In this case, the E_00_ = 2 meV, and the thermal energy KT (K is the Boltzmann constant and T = 300 K) is 25 meV; consequently, E_00_ << KT, which means that the thermionic mechanism prevails over the tunneling process [69,70,71].

**Figure 10:**
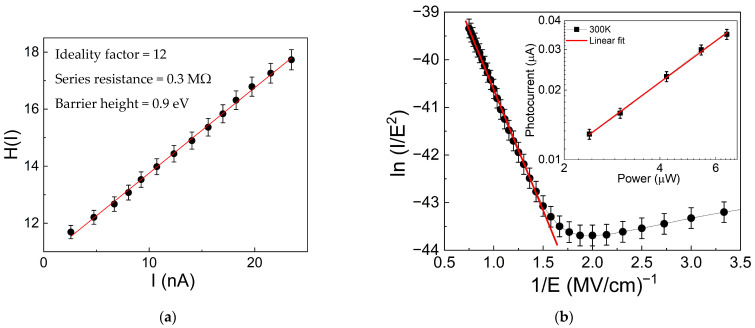
(**a**) *H*(*I*) vs. *I* curve with a linear fit (red line) to estimate the thermionic model parameters: ideality factor, series resistance, and SHB; (**b**) ln(I/E^2^) vs. E^−1^ with a linear fit (red line) to extract the Fowler–Nordheim barrier; (inset) photocurrent vs light power, with a linear fit showing a slope of one that confirms the TFL mechanism due to the presence of traps.

Generally, according to the Cheung–Cheung method, the current in the TE model can be expressed as $I=I_{o}\cdot \exp\left(\frac{q(V-IR_{s})}{nkT}\right)$, where *n* is the ideality factor, *R_s_* is the series resistance, *V* is the applied voltage, and *I*_0_ is expressed as $I_{0}=A\cdot A^{*}T^{2}exp\left(\frac{-q\phi_{B}}{kT}\right)$, in which *A* is the effective diode area, A* is the Richardson constant, and ϕ_B_ is the Schottky barrier height (SBH) [72]. Using this method, the value of the series resistance, the ideality factor, and the SBH were calculated using the following relations:

(3) $$\frac{dV}{d(lnI)}=n\left(\frac{KT}{q}\right)\;+\;IR_{s}$$

(4) $$H\left(I\right)=V-n\left(\frac{KT}{q}\right)ln\left(\frac{I}{AA^{*}T^{2}}\right)$$

(5) $$H\left(I\right)=IR_{s}-n\Phi_{B}$$

from the *H-I* plot in Figure 10a, the ideality factor, series resistance, and SBH were determined to be 12 ± 2, 0.3 ± 0.1 MΩ, and 0.9 ± 0.2 eV, respectively. The SBH value represents the Schottky barrier across the rGO/Si_3_N_4_/p-Si heterojunction of the device. It can be noted that the ideality factor is much greater than unity; this result can be attributed to the presence of a thick nitride layer between silicon and the graphene oxide [67,73,74]. It is reasonable to expect an ideality factor greater than unity, since the device is not a conventional diode-like p-n junction but a heterojunction that involves an insulator layer.

Regarding the trend of dark current for V > V_trans_, the linear relationship between ln(*I*/*E*^2^) and 1/*E*, as shown in Figure 10b, indicates the presence of F-N tunneling. From the slope of the linear fit (red line), a barrier height of approximately 0.23 ± 0.03 eV was estimated. This result suggests that the barrier height of the F-N mechanism depends on temperature, being lower at 300 K than at 77 K, consistent with the literature [75]. It shows that, at room temperature, tunnel charges through a lower triangular barrier height than in F-N tunneling at low temperature. Consequently, at 300 K, the device exhibits much higher dark current (see Figure 4b), which for V > V_trans_ displays a slope greater than two, attributed to TFL mechanisms caused by traps, confirmed by the exponent *m* = 1 obtained from the linear fit of log(*I_Ph_*) versus log(*P*), as shown in the inset of Figure 10b.

The analysis of the transport mechanism of the dark current at 77 K and 300 K reveals that the main difference occurs at low voltage (V < V_trans_): at low temperature, the hopping process dominates, whereas at room temperature, thermionic emission prevails. The transport charge for these two processes differs. In the thermionic process, charges have enough thermal energy to overcome the heterojunction barrier more easily than in the hopping process, where charges can only transit via tunneling through trapping sites because their energy is lower than the barrier. As a result, the dark current is expected to be lower at low temperatures than at room temperature, as shown by the experimental results in Figure 4b. Additionally, the F-N tunnel at V > V_trans_ shows two distinct barrier heights, 0.23 eV at 300 K and 0.9 eV at 77 K. These are correlated with the two transition bias voltages V_trans_ in the I–V curves (6 V at 300 K and 10 V at 77 K, respectively), since it is reasonable to assume that the electric field required to initiate F-N tunneling is lower for a 0.23 eV barrier than for a 0.9 eV barrier.

**Figure 11:**
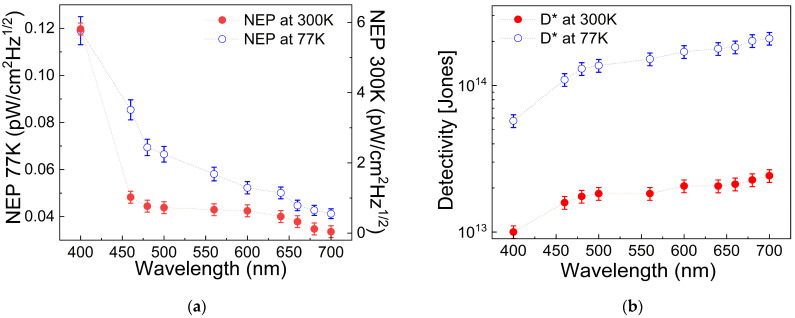
(**a**) Noise equivalent power (NEP) and (**b**) specific detectivity (D*) as a function of wavelength.

**Figure 12:**
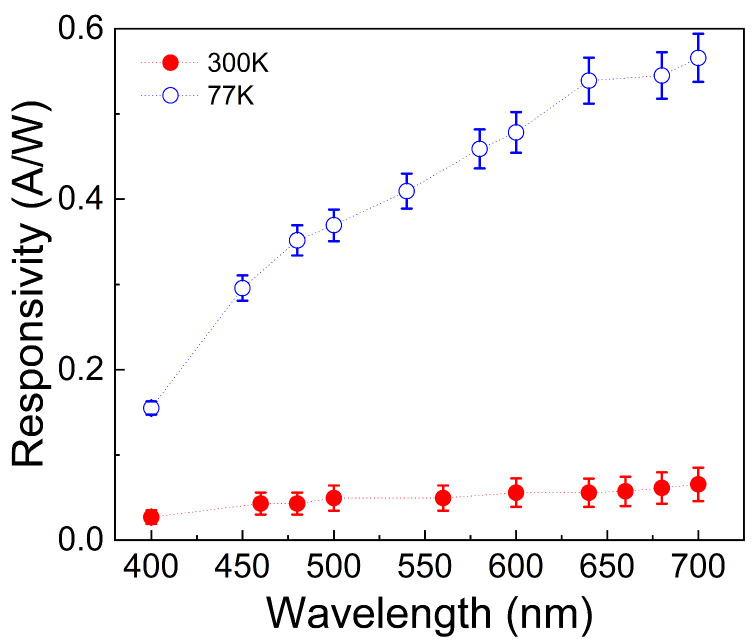
Responsivity versus wavelength of the rGO/Si_3_N_4_/p-Si device.

As mentioned earlier, the dark current intensity affects the device’s photodetection performance, and this effect can be quantified using figures of merit. In Figure 11, the noise equivalent power (NEP in Figure 11a) and the specific detectivity (D* in Figure 11b) are provided. The first parameter, $NEP=\frac{A*\sqrt{2e∆fI_{d}}}{R}$, is defined as the minimum measurable power detectable by the device, where *A* is the device’s area, *e* is the elementary charge, Δ*f* is the detection bandwidth of 1 Hz, *I_d_* is the dark current density, and $R=\frac{I_{Ph}}{P}$ is the responsivity, with *I_Ph_* and the *P* representing the photocurrent and the light power, respectively [35]. Conversely, D* = A^1/2^/NEP indicates the device’s ability to detect low-light levels [76]. It is notable that at 77 K, the NEP is lower than at 300 K, and D* is improved. As a result, the device’s responsivity (shown in Figure 12) at low temperature exceeds that at room temperature, confirming that low dark current enhances photodetection efficiency by minimizing charge recombination and current leakage.

The improvement in responsivity at low temperature demonstrates that the photogenerated charges are efficiently collected at the electrodes, thanks to reduced thermionic processes that could otherwise induce current leakage. It should be noted that the increase in responsivity occurs over the broadband range from 400 nm to 700 nm, so it is reasonable to assert that this result does not depend on the possible effect of the rGO gap temperature dependence.

Table 1 summarizes the differences between the features of the rGO-based device at 300 K and 77 K. The experimental results show temperature-dependent behavior in specific characteristics, including the bias transition voltage, the transport mechanism at low voltages, the F-N barrier height, and the photodetection figure of merit. The results indicate that at room temperature, the heterojunction rGO/Si_3_N_4_/p-Si performs worse in terms of the photodetection figure of merit than rGO/Si_3_N_4_/n-Si, as reported in ref. [35]. Conversely, at 77 K, the rGO/Si_3_N_4_/p-Si exhibits superior performance compared with its n-type silicon counterpart. Therefore, photodetectors based on an rGO layer deposited on p-type silicon could be suitable for applications requiring low-temperature conditions, i.e., satellite surveillance of the territory for environmental security purposes, sensors for telescopes for astrophysical studies.

It is worth noting that the temperature-dependent analysis reported in this work, which is not limited to the rGO layer or rGO/Si junction, as previously reported in the literature, demonstrates the device’s photodetection performance at cryogenic temperatures. The findings, from a physical perspective, explain the relationship between the dark current and photogeneration efficiency and highlight the role of quantum processes in the photodetection capability of rGO/silicon-based heterojunctions. These results, reproduced across devices based on the rGO/Si_3_N_4_/p-Si heterojunction, could serve as a roadmap for identifying the most critical parameters, such as dark current and heterojunction structure, to be considered when developing photodetectors with desired detection capabilities. Furthermore, enhancements to the room-temperature performance could be achieved by protecting the rGO layer from the effects of air humidity, which reduces its degree, and by minimizing light reflection loss at the rGO surface.

## 5. Conclusions

In this work, photodetectors based on rGO/Si p-type junctions were developed. The results indicate that photodetectors based on reduced graphene oxide exhibit notable performance, particularly at low temperatures. Optoelectronic characterization of the rGO layer reveals that the material consists of micrometer-sized flakes and exhibits semiconductor-like electronic behavior, with an optical band gap of approximately 2.8 eV. A key finding for the device based on the rGO/Si_3_N_4_/p-Si heterojunction is a reduction in dark current at 77 K. This was investigated by analyzing the electronic transport mechanisms active at 77 K and 300 K. The results demonstrate that the low-temperature hopping mechanism yields low losses and minimal dark current.

Additionally, the F-N tunneling process emphasizes the device’s quantum features, whereas the threshold voltage defines the photodetector’s typical on-off states. Lastly, the enhanced photodetection performance at 77 K was confirmed by estimating figures of merit, including NEP, Detectivity, and Responsivity, over the wavelength range from 400 nm to 700 nm. The rGO/Si_3_N_4_/p-Si heterojunction device shows potential as a UV-Vis-NIR light detector suitable for low-temperature applications such as astronomical observation and military defense, where reliable performance at low temperatures, long-term stability, and low cost are required.

## Figures and Tables

**Table 1:** Summary of the main parameters for the rGO-based device at 300 K and 77 K. V_trans_, F-N, and TFL represent the transition bias voltage, the Fowler–Nordheim tunneling mechanism, and the trap-filled limit mechanism, respectively.

Temperature (K)	V_trans_ (V)	Transport Mechanism	F-N Barrier Height (eV)	Responsivity (A/W) ^1^ 400 nm–700 nm
300	6	Thermionic and F-N + TFL	0.23 ± 0.03	0.03–0.07
77	10	Hopping and F-N + TFL	0.9 ± 0.1	0.17–0.56

^1^ In the table, only the maximum and minimum responsivity values are reported.

## Data Availability

The original contributions presented in this study are included in the article. Further inquiries can be directed to the corresponding author.
